# Predicting mortality risk in dialysis: Assessment of risk factors using traditional and advanced modeling techniques within the Monitoring Dialysis Outcomes initiative

**DOI:** 10.1111/hdi.13053

**Published:** 2022-11-20

**Authors:** Sheetal Chaudhuri, John Larkin, Murilo Guedes, Yue Jiao, Peter Kotanko, Yuedong Wang, Len Usvyat, Jeroen P. Kooman

**Affiliations:** ^1^ Fresenius Medical Care Global Medical Office Waltham Massachusetts USA; ^2^ Maastricht University Medical Center Maastricht The Netherlands; ^3^ Pontifícia Universidade Católica do Paraná Curitiba Brazil; ^4^ Renal Research Institute New York New York USA; ^5^ Icahn School of Medicine at Mount Sinai New York New York USA; ^6^ University of California Santa Barbara California USA

**Keywords:** clinical risk factors, machine learning model, MONDO database, mortality prediction

## Abstract

**Introduction:**

Several factors affect the survival of End Stage Kidney Disease (ESKD) patients on dialysis. Machine learning (ML) models may help tackle multivariable and complex, often non‐linear predictors of adverse clinical events in ESKD patients. In this study, we used advanced ML method as well as a traditional statistical method to develop and compare the risk factors for mortality prediction model in hemodialysis (HD) patients.

**Materials and Methods:**

We included data HD patients who had data across a baseline period of at least 1 year and 1 day in the internationally representative Monitoring Dialysis Outcomes (MONDO) Initiative dataset. Twenty‐three input parameters considered in the model were chosen in an a priori manner. The prediction model used 1 year baseline data to predict death in the following 3 years. The dataset was randomly split into 80% training data and 20% testing data for model development. Two different modeling techniques were used to build the mortality prediction model.

**Findings:**

A total of 95,142 patients were included in the analysis sample. The area under the receiver operating curve (AUROC) of the model on the test data with XGBoost ML model was 0.84 on the training data and 0.80 on the test data. AUROC of the logistic regression model was 0.73 on training data and 0.75 on test data. Four out of the top five predictors were common to both modeling strategies.

**Discussion:**

In the internationally representative MONDO data for HD patients, we describe the development of a ML model and a traditional statistical model that was suitable for classification of a prevalent HD patient's 3‐year risk of death. While both models had a reasonably high AUROC, the ML model was able to identify levels of hematocrit (HCT) as an important risk factor in mortality. If implemented in clinical practice, such proof‐of‐concept models could be used to provide pre‐emptive care for HD patients.

## INTRODUCTION

There are several factors affecting the survival of End Stage Kidney Disease (ESKD) patients on dialysis. In addition to demographic factors such as age and gender, fluid overload, inflammation, serum phosphate levels, indications of malnutrition and loss of lean tissue mass assessed by bioimpedance spectroscopy (BIS) have a strong association with clinical outcomes.^1^, ^2^, ^3^, ^4^, ^5^ Adding to the complexities related to such multiple prognostic markers, many of them have a bimodal relation with clinical outcomes and are also dependent on the interaction with other parameters. For example, blood pressure, inter‐dialytic weight gain (IDWG), and phosphate have non‐linear, bimodal associations with worse outcomes. Also illustrative, the association between systolic blood pressure and clinical outcomes is modified by the fluid status, whereas the associations between phosphate and clinical outcomes are influenced by nutritional state.^6^, ^7^ While we know inflammatory, nutritional, body composition, and fluid overload markers are associated to clinical outcomes in hemodialysis (HD) patients, they have not been studied together in a multi‐modal analysis using advanced analytical models. Furthermore, current risk prediction models lack detailed assessments of fluid state and body composition.

There are several differences between traditional statistical methods vs advanced analytical techniques.^8^ Traditional statistical methods are easily interpretable, while advanced machine learning (ML) techniques are powerful at analyzing complex data formats such as audio or images. Advanced ML models can deal with such non‐linear relationships, efficiently handle missing data, and thus could be used to enhance risk prediction models in HD patients.

The goal of this study is to understand if it is feasible to develop a prediction model to predict mortality in HD patients using a large globally representative dialysis database with clinical parameters such as nutritional, inflammatory, hydration, anemia, and mineral metabolism related parameters collected consistently across different regions in various electronic medical records (EMR). In this effort, we also assessed the application of advanced analytical ML method as well as simple traditional statistical method and highlighted how the outputs of the two approaches are similar and/or different. We further attempted to explain the results of the advanced ML model while comparing it to the output of the traditional model which has been generally well understood in the clinical community.

## METHODS/DESIGN

### Patient cohort

This was a retrospective observational cohort study that used the Monitoring Dialysis Outcomes (MONDO) Initiative dataset.^9^, ^10^ We included data from all unique HD patients who had data across a baseline period of at least 1 year and 1 day (i.e., ≥2 HD treatment records ≥1 year & 1 day apart). The follow‐up period for the assessment of mortal outcomes was up to 3 years. The research activities conducted in the MONDO Initiative comply with all applicable national and international ethical standards. Western Institutional Review Board approved a protocol on the MONDO initiative study projects and determined analyses are exempt due to use of de‐identified data (Work Order 1–939512‐1). Furthermore, the MONDO dataset has had a re‐identification risk assessment performed by Privacy Analytics and has been fully anonymized using techniques that satisfy the concept of anonymization by the European Union General Data Protection Regulation (GDPR) and satisfy the concept of de‐identification by the United States Department of Health and Human Services (HHS). This study was performed in adherence with the Declaration of Helsinki.

### Model data and features

**FIGURE 1:**
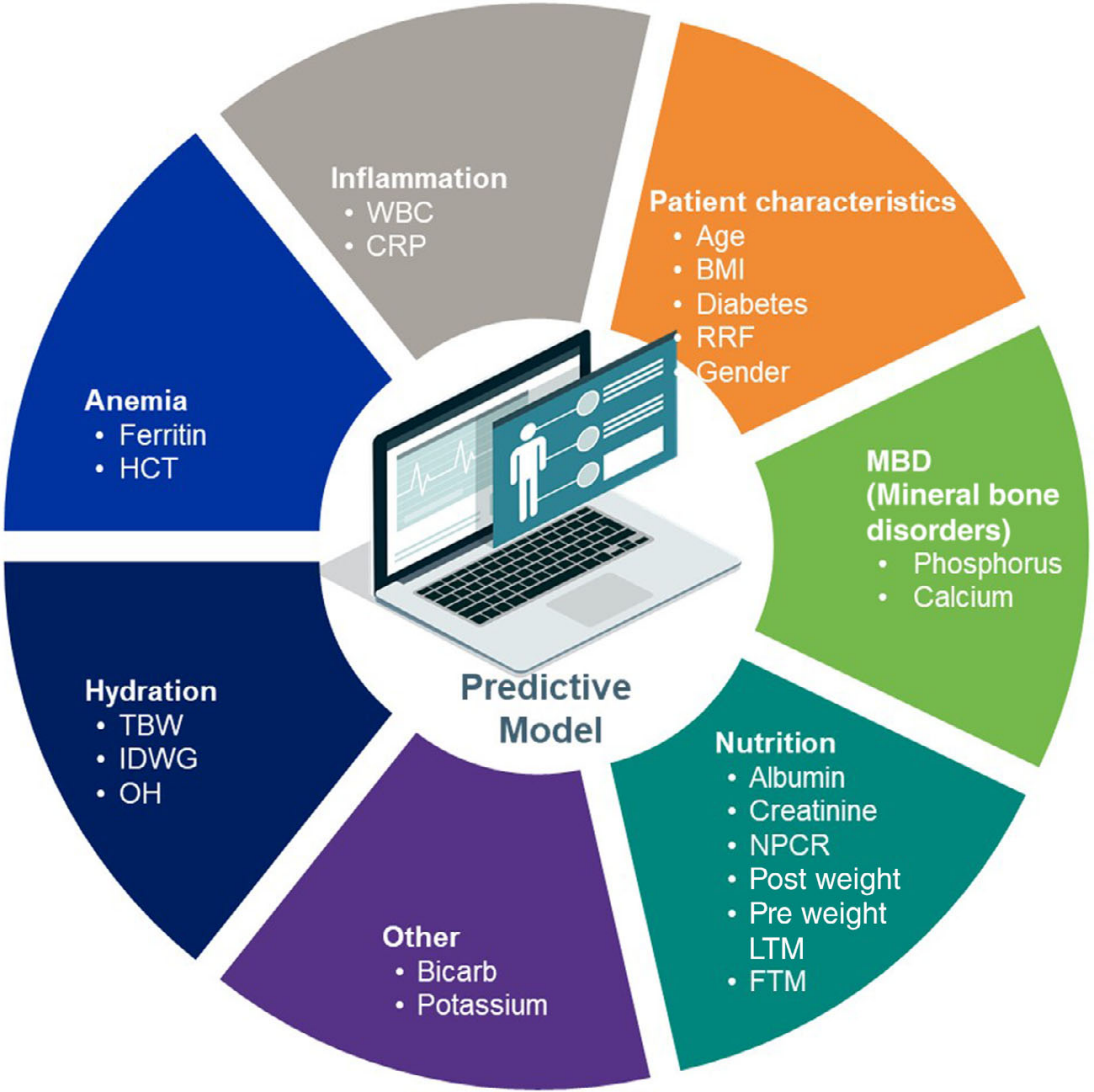
Groups of input parameters [Color figure can be viewed at wileyonlinelibrary.com]

The outcome of all‐cause mortality (dependent variable) was recorded during a 3‐year follow‐up period after baseline. The clinical data were collected per the standard practices in each country. While most patients in regions receive HD treatment thrice a week, it could vary from region to region. Only those input parameters that were consistently captured across regions were chosen in an a priori manner and considered in the model (independent variables). Patient characteristics such as age and body mass index (BMI) were calculated as the maximum recorded value during the baseline period. BMI was calculated by dividing maximum of the post HD weight and the square of maximum height in meters. Laboratory and clinical parameters such as albumin, normalized protein catabolic rate (NPCR), bicarbonate, calcium, creatinine (CREAT), c reactive protein (CRP), ferritin, hematocrit (HCT), phosphate, potassium, pre‐HD weight, post‐HD weight, white blood cell (WBC) count, and residual renal function (RRF) were derived from the average value during the baseline period. RRF was captured as the glomerular filtration rate determined by urine collection. BIS measurements such as fat tissue mass (FTM), lean tissue mass (LTM), and total body water (TBW) were also averaged over the baseline period. IDWG was calculated as the difference between pre‐HD weight at the index treatment and post‐HD weight at the prior treatment, and the IDWG values were averaged over the baseline period. Overhydration (OH) was calculated as the difference between pre‐weight and normal hydration weight measured by the BIS, and were averaged over the baseline period. Figure 1 shows categories of various input parameters used in the model.

### Predictive model

**FIGURE 2:**
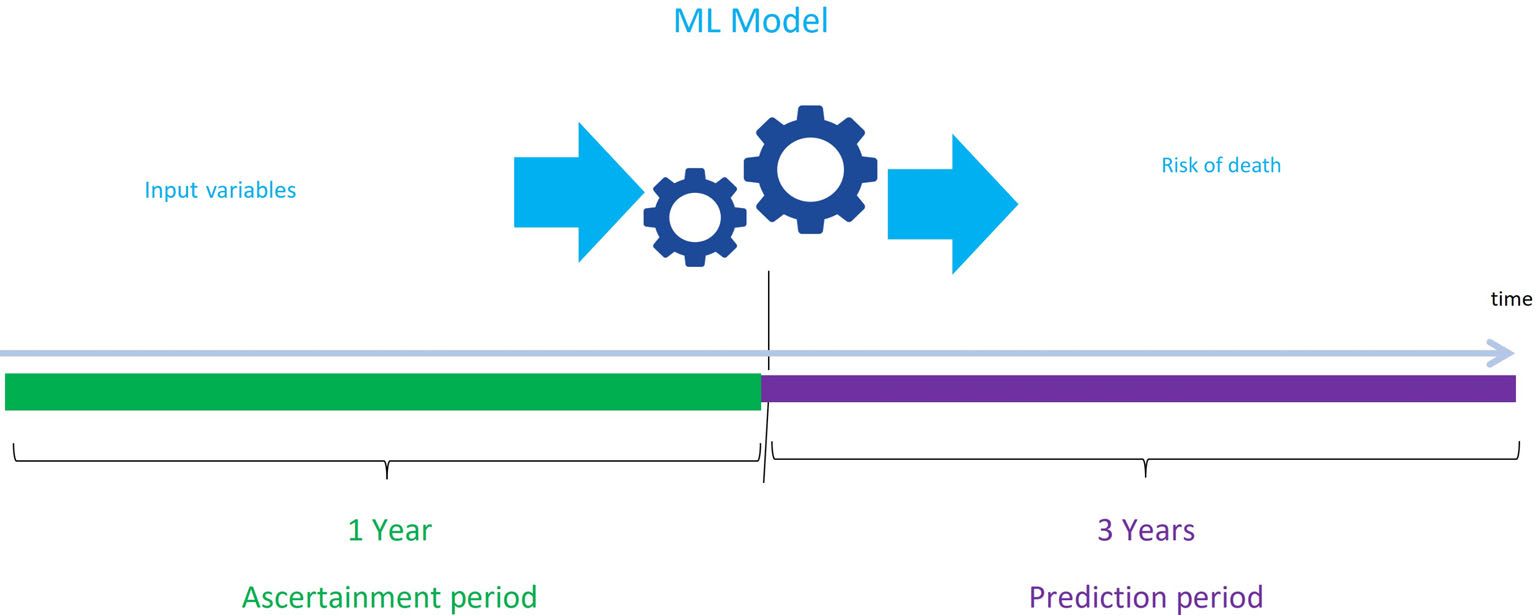
Ascertainment period and prediction period [Color figure can be viewed at wileyonlinelibrary.com]

The prediction model used 1 year baseline data for incenter HD patients to predict death in the following 3 years. Figure 2 shows the ascertainment period and prediction period of the advanced ML and logistic regression prediction model. The model only predicts mortality for patients who survived and have data for at least 1 year. The cohort dataset was randomly split into 80% training data and 20% testing data for model development.

### Advanced analytical model development

Python version 3.7.7 (Python Software Foundation, Delaware) was used to develop the advanced ML model utilizing the XGBoost package.^11^ The XGBoost Python package uses input parameters based on the training dataset to construct decision trees. Each decision tree provided a random sample and a series of thresholds that split parameters to maximize information gain. These decision trees are created iteratively and new decision trees are developed to minimize prior prediction errors.^12^ The decision trees made by the XGBoost ML model can handle missing values without imputation by recognizing their presence when determining the splits. The ML model was constructed using the training data (80% of the cohort) and the final model was assessed using the unseen testing data (20% of the cohort).

### Traditional analytical model development

SAS 9.4 was used to build the logistic regression model. Stepwise logistic regression model (slentry = 0.3 and slstay = 0.35) was developed using the same input parameters and the training data as the advanced ML model.^13^

Imputation in the form of mean or median was used to fill in data where it was incomplete. The stepwise logistic regression model was also tested on the 20% test data.

### Analysis of ML and logistic regression models performance

Performance of the advanced ML model and the logistic regression model was evaluated by the area under the receiver operating curve (AUROC) in the training and testing datasets.^14^

### Analysis of feature importance advanced ML model

Shapley values are calculated using the SHAP Python package to define the effect of each parameter on the predictions.^15^, ^16^ SHAP values are computed for each input parameter, representing a measure of effect (positive or negative value) of the input parameter on each individual prediction. SHAP methods withhold and include all combinations of individual input parameters and then compare differences between withheld and included data. Mean value of all possible differences is then used to calculate the feature importance. SHAP values are additive explanations of feature importance and are presented as log odds (i.e., the logarithm of the odds ratio). SHAP values for each set of observations are summed, and converted from log odds to probability, which is then output by the model as the prediction. Positive SHAP values increase the predicted probability, whereas negative SHAP values decrease the predicted probability.

Partial dependence plots (PDP) were created using the SHAP values to analyze the bimodal associations between two parameters and the impact on mortality. It shows the localized effects of two parameters on the predicted outcome. It also shows whether the associations are linear or more complex.

### Analysis of feature important in traditional model

The summary statistics from the logistic regression model shows a list of the input parameters from the training dataset in the order of importance. *p*‐value of <0.05 was considered statistically significant. Odds Ratio Estimates (OR) estimate from the stepwise logistic regression model shows the association between the risk factor and outcome. It represents the odds or the probability of the risk factor altering the predicted outcome.^17^

## RESULTS

### Patient characteristics

**FIGURE 3:**
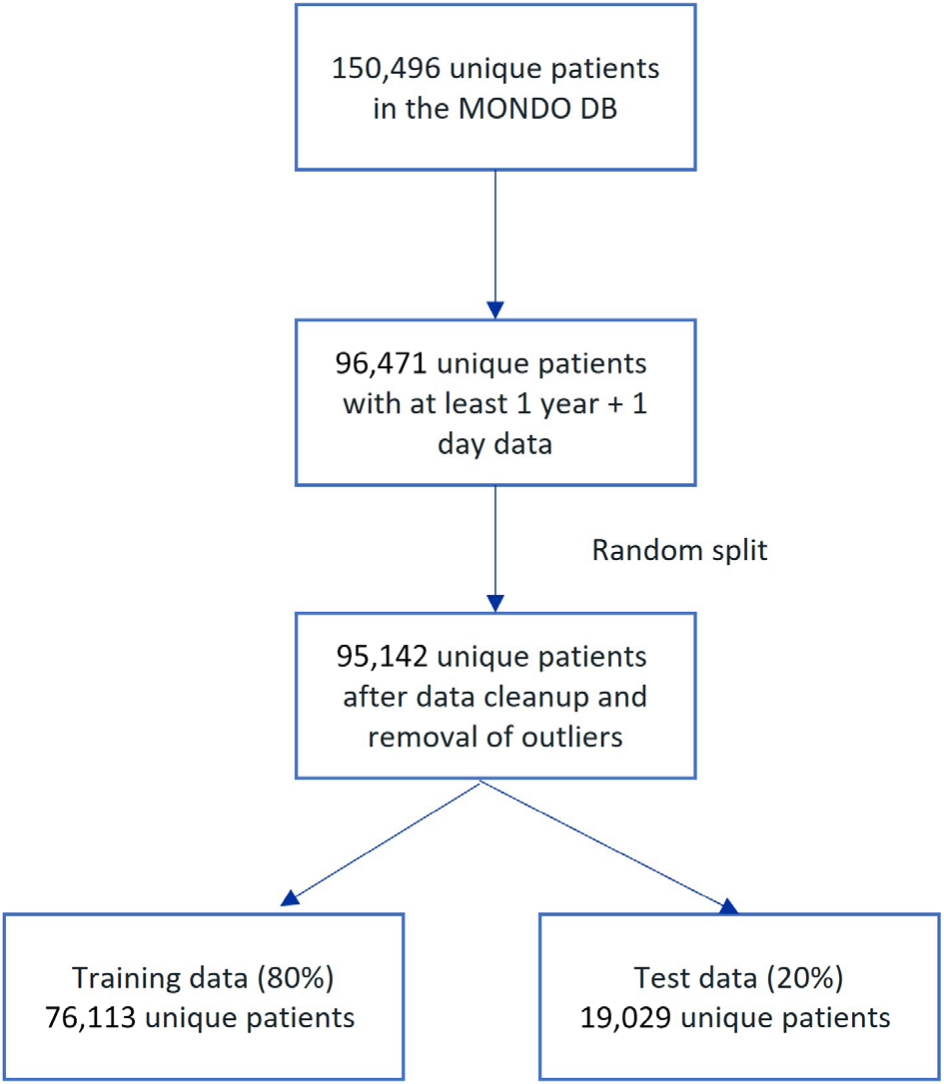
Flow diagram of study data [Color figure can be viewed at wileyonlinelibrary.com]

Out of 150,496 unique patients in the MONDO Initiative dataset, 95,142 patients who had data recorded across a baseline period of 1 year and 1 day were included and assessed during a 3‐year follow‐up period. Figure 3 shows a flow diagram of the data used in the study. The overall follow‐up time was an average 2.8 years. Among patients who died, the average follow‐up time was 1.34 years. Among those who survived, the average follow‐up time was 3 years. The majority were male (57.4%) with an average age of 61.7 years and 62% of them were diagnosed with diabetes mellitus. Table 1 shows the regional spread of the 95,142 patients. Table 2 shows the descriptive statistics of the numeric input parameters during the baseline period.

**TABLE 1:** Distribution of patients by region

Region	Count	Percentage
Southern Europe	20,178	21.21%
Eastern Europe	19,705	20.71%
South America	17,530	18.43%
Eastern Asia	12,402	13.04%
Western Asia	9465	9.95%
Northern Europe	7046	7.41%
Northern America	4419	4.64%
Western Europe	2825	2.97%
Southeastern Asia	805	0.85%
Oceania	731	0.77%
Other	36	0.04%
Total worldwide	95,142	100%

**TABLE 2:** Descriptive statistics of numeric input parameters

	Count	Mean	Std	Min	25%	50%	75%	Max
Albumin (g/L)	52,099	3.78	0.42	1.36	3.54	3.81	4.05	6.00
NPCR (g/kg/day)	37,324	1.00	0.24	0.01	0.92	1.01	1.08	22.64
BICARB (mEq/L)	22,361	22.41	2.86	2.00	20.67	22.43	24.20	45.00
Calcium (mg/dl)	43,821	8.86	0.64	4.01	8.48	8.84	9.21	14.92
CREAT (mg/dl)	44,172	7.40	2.48	0.30	5.67	7.15	8.84	25.00
CRP (mg/dl)	39,576	11.07	16.12	0.10	1.65	5.10	13.35	160.00
Ferritin (ng/ml)	52,340	418.08	257.85	2.00	230.72	370.03	551.40	1650.00
HCT (%)	38,380	34.19	3.62	15.00	32.14	34.39	36.50	53.00
PHOSPH (mg/dl)	54,362	4.27	1.59	1.00	3.47	4.46	5.27	18.29
Postweight (kg)	46,141	69.95	16.49	22.35	58.51	67.97	78.93	212.12
Preweight (kg)	46,284	71.95	16.73	31.70	60.38	69.96	81.09	214.40
Potassium (mEq/L)	54,151	4.87	0.62	2.15	4.45	4.84	5.26	8.70
IDWG (kg)	46,128	1.99	0.83	0.00	1.43	1.95	2.48	14.38
WBC (1000/mc)	35,527	6.91	2.72	0.00	5.63	6.81	8.13	100.00
RRF (ml/min)	3168	5.77	5.75	0.00	2.48	4.40	8.00	97.00
BSI FTM (%)	2297	22.72	10.08	5.06	15.11	21.43	29.04	54.39
BSI LTM (%)	2308	46.96	6.09	40.00	42.47	45.29	49.70	84.50
BSI TBW (%)	2345	41.83	5.15	30.11	38.20	41.21	44.84	71.62
Age (years)	64,313	61.73	15.08	18.00	52.00	64.00	73.00	90.00
BMI (kg/m^2^)	18,706	25.13	5.53	11.68	21.49	24.22	27.63	83.19
OH (kg)	2334	1.36	3.11	−14.74	−0.11	1.49	2.90	14.65

Abbreviations: BICARB, bicarbonate; BIS, bioimpendance spectroscopy; BMI, body mass index; CREAT, creatinine; CRP, C reactive protein; FTM, fat tissue mass; HCT, heamtocrit; IDWG, inter‐dialytic weight gain; LTM, lean tissue mass; NPCR, normalized protein catabolic rate; OH, OverHydration = PreWeight − normal hydration weight (BIS Measurement); RRF, residual renal function; TBW, total body water; WBC, white blood cell.

### ML model performance and feature importance

**FIGURE 4:**
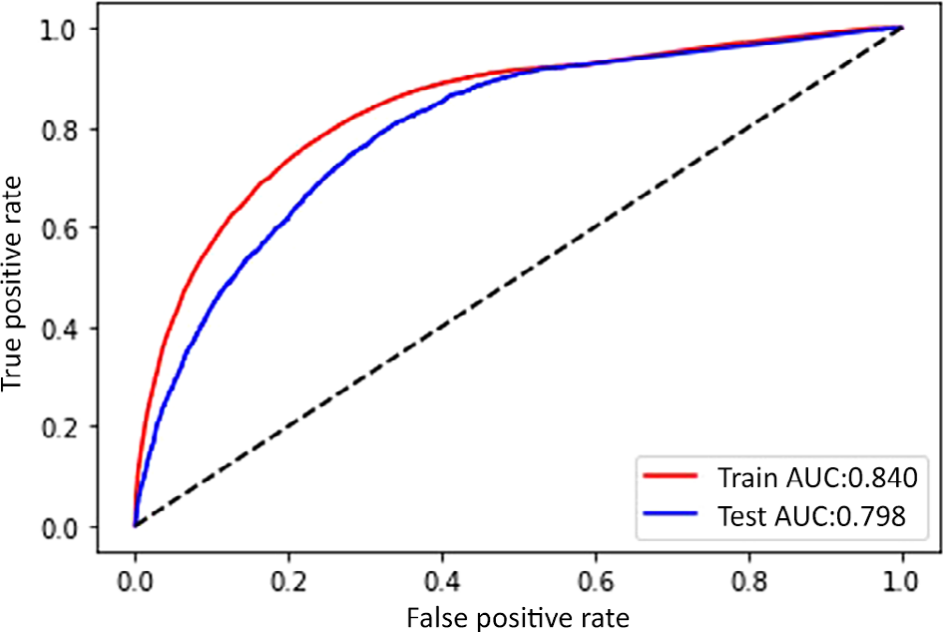
Area under the curve for XGBoost model [Color figure can be viewed at wileyonlinelibrary.com]

**FIGURE 5:**
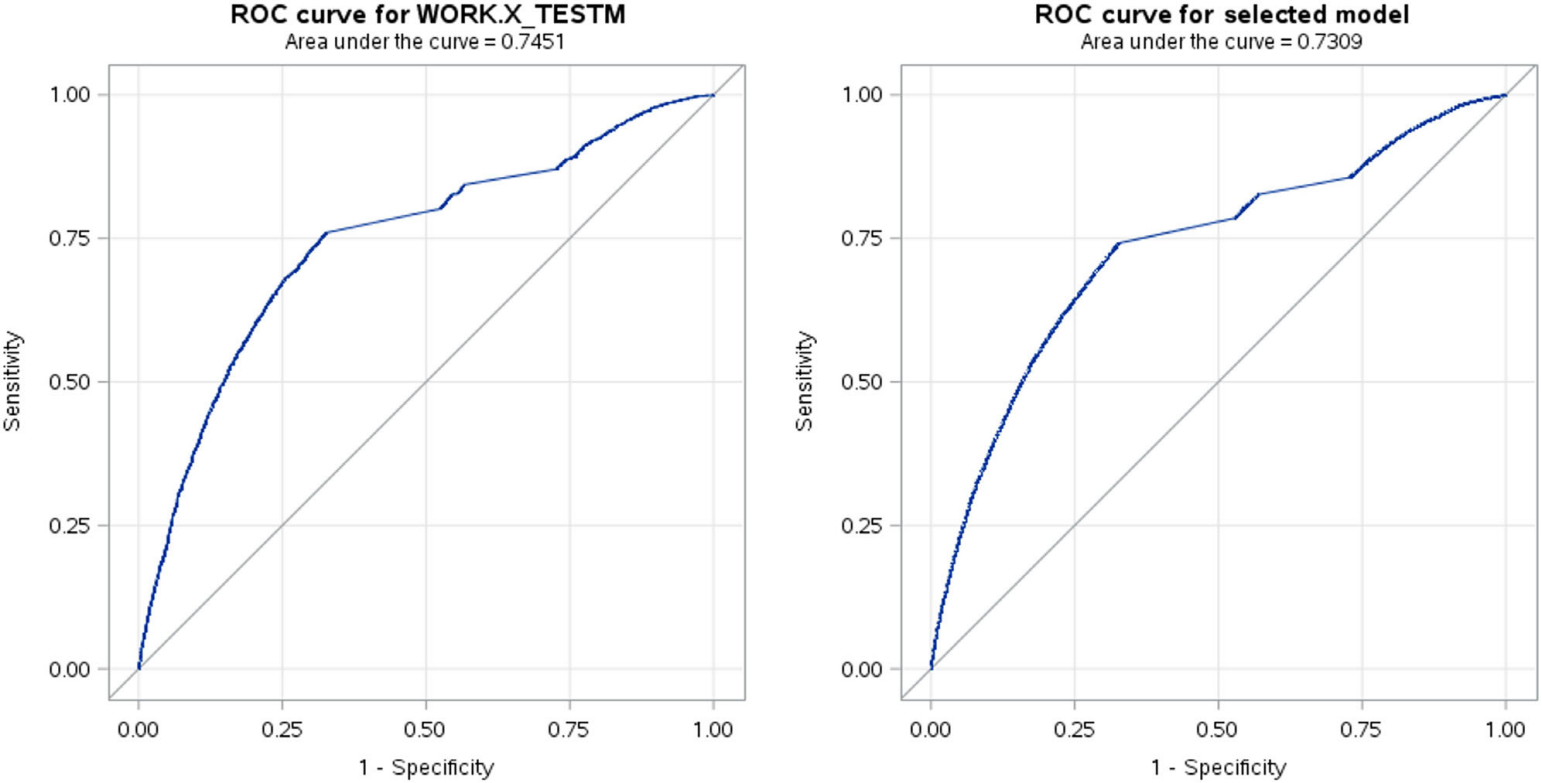
Area under the receiver operating curve(AUROC) for logistic regression model with training and test data [Color figure can be viewed at wileyonlinelibrary.com]

The resulting advanced analytical predictive model was tested on 20% of the patient's data, which was withheld and unseen during training. The AUROC of the model on the test data with XGBoost ML model was 0.84 on the training data and 0.80 on the test data. Figure 4 shows the AUROC of the XGBoost ML model. Similarly, Figure 5 shows the AUROC curve from the logistic regression model. It was 0.73 on the training and 0.75 on the test data.

### Important features using SHAP values

**FIGURE 6:**
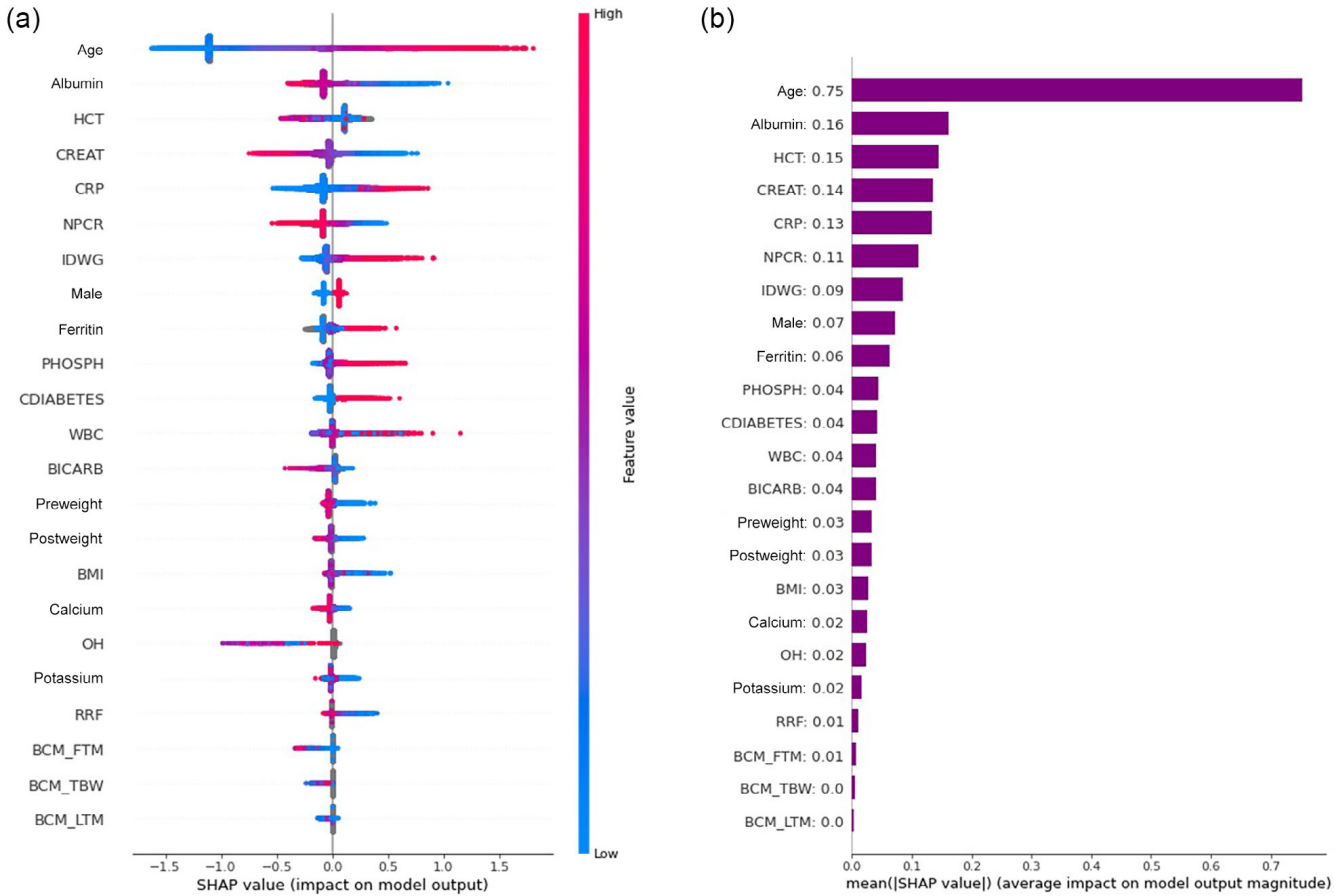
Predictors of death from XGBoost model shown in descending order. (a) SHAP value plots show the size and direction (more positive = higher risk or more negative = lower risk) of each variable's influence on the outcome for each unique patient on the *x*‐axis, with warmer colors representing higher observed values for that measurement, cooler colors indicating lower values for that measurement, and gray representing a missing value for that measurement. SHAP values are presented in the unit of log odds (i.e. logarithm of the odds ratio). (b) Absolute value of the average SHAP value shows on average how much each predictor impacts the mortality prediction [Color figure can be viewed at wileyonlinelibrary.com]

Figure 6a shows the magnitude of the SHAP value for the top predictors identified by the XGBoost Model in a descending order of importance. Warmer colors on the figure show a higher impact of the parameter in predicting mortality, while cooler colors show a the negative (i.e., protective) impact of the parameter on predicting mortality. Patients with higher age, lower HCT, lower albumin, lower CREAT, higher CRP, lower NPCR, higher IDWG, higher ferritin, higher phosphorous, higher WBC, presence of diabetes, lower potassium, lower BMI, low RRF, and lower pre and post weight have a higher chance of mortality in the following 3 years. Similarly, higher CREAT, higher HCT, higher NPCR, higher BICARB, lower overhydration, higher BIS measurements of FTM, lower LTM, and slightly higher TBW have a lower chance of mortality in the following 3 years. Figure 6b shows the absolute value of the average SHAP value that is the value shows on average how much each predictor impacts the mortality prediction either in the positive or negative direction.

**FIGURE 7:**
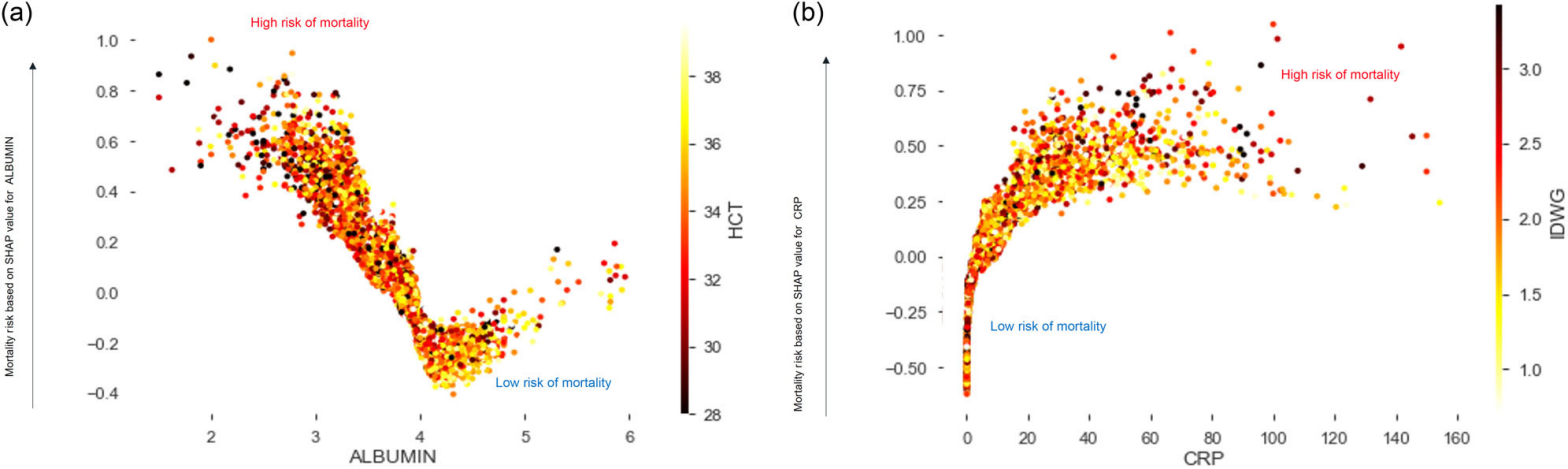
Partial dependence plots: Each dot corresponds to an individual person in the study. The dot's position on the *x*‐axis shows the impact that parameter has on the model's prediction for that person. Multiple dots in the same position show density. Risk of mortality based on the SHAP value of the parameter on the *X* axis is shown on the *Y* axis. (a) Partial Dependence Plots for Nutrition Related Parameters; (b) Partial Dependence Plots for Hydration Related Parameters [Color figure can be viewed at wileyonlinelibrary.com]

Figure 7a,b show a couple of PDP plots for individual parameters used in the model. These plots help understand the interaction of select top predictors in predicting mortality among different domains shown in Figure 1. Each dot corresponds to an individual person in the study. The dot's position on the *X*‐axis shows the impact that parameter has on the model's prediction for that person. Multiple dots in the same position show density. Risk of mortality based on the SHAP value of the parameter on the *X*‐axis is shown on the *Y*‐axis.

The first PDP plot shows the association between a selected nutrition related parameter (albumin) and an anemia related parameter (HCT). The graph shows the associations between variables, as well as the impact on mortality. Patients with lower albumin and lower HCT have the highest risk of mortality predicted as shown by the dark red dots on the top left corner of Figure 7a. Patients with higher albumin and higher HCT have the lowest impact on mortality as shown by the cluster of light‐yellow colored dots on the bottom right corner of the plot.

The second PDP plots show the association between a selected inflammation related parameter (CRP) and a hydration related parameter (IDWG) on mortality. Higher CRP and higher IDWG have a higher impact on mortality as indicated by the dark red color clusters in the top righthand corner of the graph. Patients with lower CRP have lower risk of mortality regardless of the IDWG.

### Results from the logistic regression model

Table 3 shows the parameters from the stepwise logistic regression model in a descending level of significance along with the odds ratio estimate and 95% confidence interval. The results show most of the input parameters other than LTM, FTM, TBW, HCT, and pre‐weight have a statistically significant (*p* < 0.05) impact on the output of the logistic regression model. Male gender, higher age, higher levels of CRP, ferritin, phosphorous, IDWG, WBC, RRF, LTM, OH, and BMI associate with an increased risk of mortality in the follow‐up period. The assessment between the directionality provided by the odds ratio estimates and the SHAP values is presented by the color coding in Table 3. The odds ratio estimates shown in red font indicate the risk of mortality increases with the increasing value of the input parameter in a consistent manner with the results shown in the SHAP values from the XGBoost model. The odds ratio estimates shown in a blue font indicate the risk of mortality decreases with increasing value of the input parameter in a consistent manner with the results shown by SHAP values. The odds ratio estimates without any color indicate that the directionality of mortality risk in the SHAP value and the logistic regression are not the same.

**TABLE 3:** Summary of step‐wise selection and odds ratio estimate

Step	Entered	Chi‐Square	Significance	Odds ratio	95% confidence limits	
1	Age	2743.08	<0.0001	1.050	1.048	1.053
2	CRP	901.22	<0.0001	1.017	1.015	1.019
3	Albumin	560.65	<0.0001	0.486	0.452	0.523
4	CREAT	367.45	<0.0001	0.835	0.822	0.849
5	CDIABETES	431.76	<0.0001	0.555	0.517	0.596
6	PHOSPH	190.47	<0.0001	1.129	1.106	1.154
7	IDWG	129.52	<0.0001	1.255	1.204	1.308
8	Ferritin	59.04	<0.0001	1.000	1.000	1.001
9	Male	43.28	<0.0001	1.18	1.124	1.239
10	NPCR	25.82	<0.0001	0.406	0.323	0.511
11	BICARB	12.84	0.000	0.970	0.955	0.986
12	BMI	11.23	0.000	1.030	1.020	1.041
13	Postweight	33.08	<0.0001	0.993	0.990	0.995
14	WBC	7.52	0.006	1.018	1.006	1.031
15	OH	7.2225	0.0072	1.094	1.038	1.154
16	Potassium	4.0704	0.0436	0.949	0.903	0.998
17	Calcium	3.9427	0.0471	0.95	0.902	1.001
18	RRF	3.7162	0.0539	1.016	0.999	1.033
19	BCM_TBW	1.9393	0.1637	0.956	0.913	1.001
20	BCM_LTM	1.5422	0.2143	1.046	1.008	1.085

*Note*: Red: Risk of mortality increases with the increasing value of the input parameter in a consistent manner with the results shown in the SHAP values from the XGBoost model; Blue: Risk of mortality decreases with the increasing value of the input parameter in a consistent manner with the results shown in the SHAP values from the XGBoost model; Black: The direction of the risk of mortality between the logistic regression model and the XGBoost model is not consistent.

Abbreviations: BICARB, bicarbonate; BMI, body mass index; CREAT, creatinine; CRP, C reactive protein; IDWG, inter‐dialytic weight gain; LTM, lean tissue mass; NPCR, normalized protein catabolic rate; OH, OverHydration = PreWeight − normal hydration weight (BIS Measurement); RRF, residual renal function; TBW, total body water; WBC, white blood cell.

## DISCUSSION

In the internationally representative MONDO Initiative cohort of hemodialysis patients from dialysis providers throughout 37 countries in 5 continents,^10^ we describe the development of a ML model and a traditional statistical model that was suitable for classification of a prevalent HD patient's 3‐year risk of death. The performance of both techniques (AUROC 0.80 for ML model 0.75 for traditional model) was high and much better than chance (i.e., AUROC 0.50). Furthermore, due to the inherent ability of the ML modeling technique to account for collinearity between input variables and handle missing data, we were able to identify important predictors for death events, such as HCT—the third most important prognostic risk factor. Importantly, the prediction model used a set of input parameters that is frequently collected in the standard care of HD patients by most dialysis providers worldwide. Thus, our proof‐of‐concept model and findings are generalizable, and the model may be scalable for prognostic decision support in the care of dialysis patients.

There have been other mortality prediction models developed in dialysis patients; however, most models have overestimated the probability of death.^18^ Table 4 references several of these studies along with the size and the geographic distribution of the cohort. The AUROC, or the C‐statistic, of most models described is lower than the AUROC of the advanced ML model described in this paper. There are a couple reports on mortality models where the AUROC was comparable with our ML model; however, they were tested on considerably smaller cohorts.^26^, ^28^ A strength of our model is it was trained and tested on a large cohort of patients geographically distributed across the globe who were treated by multiple providers.^10^

**TABLE 4:** Summary of other mortality prediction models in HD patients

Author	ML model	Number of patients/region	AUROC on test data
Siga et al.^19^	Bayesian network	9010 (Europe)	0.78
Jung et al.^20^	Cox proportional hazards regression analysis	3309 (Korea)	0.74
Zhu et al.^21^	Cox model	173 (China)	0.79
Wang et al.^22^	Long short term memory autoencoder	1200 (China)	0.57
Tapak et al.^23^	Randon survival forests	785 (Iran)	0.80
Thijssen et al.^24^	Logistic regression	6838 (North America)	0.72
Hemke et al.^25^	Cox regression analysis	1835 (Netherlands)	0.78
Doi et al.^26^	Logisitc regression	688 (Japan)	0.83
Floege et al.^27^	Cox model	11,508 (Europe)	0.73
Cohen et al.^28^	Cox model	512 (North America)	0.80
Holme et al.^29^	Cox model	868 (region not available)	0.72

Abbreviations: AUROC, area under the receiver operating curve; HD, hemodialysis.

It is worthwhile to detail the ML modeling technique we used is not based on linear relationships for the input variables. This can be advantageous since the method does not assume linearity and can avoid bias from *u*‐, *j*‐, and other irregular‐shaped associations with an independent variable and an outcome, yet the interpretability of the coefficients can be difficult since they are reported in log odds. Unlike the ML model in this study, traditional modeling models (e.g., logistic regression, Cox proportional hazards) frequently assume linearity in relationships and are unable to handle missing data. Hence, imputation of missing data becomes necessary like we performed for the logistic regression model assessed in our study. Traditional models can be easier to interpret with odds or hazards ratios providing the proportion of risk per unit of change in the parameter based off an assigned reference point. ML models are best suited for complex datasets especially with missing data.

Table 3 shows how the important variables identified by each modeling technique. Four out of the top five predictors from both models are common and display similar directionality. Patients with higher age, lower albumin, higher CRP, and lower creatinine have higher risk of mortality in the following 3‐years. Other studies have shown age, albumin and CRP are significant factors in predicting mortality in HD patients.^30^ HCT did not appear significant in the logistic regression model yet was the third most important risk factor in the ML model. Anemia is a strong predictor of worse clinical and patient‐reported outcomes, yet anemia correction has not been shown to improve clinical outcomes, except at severely low hemoglobin levels for currently available interventions.^31^, ^32^ As anemia interventions, such as iron deficiency management and erythropoietic stimulating agent prescription, may be linked to clinical outcomes regardless of hemoglobin levels, observational analysis of achieved hemoglobin levels may lead to important heterogeneity.^33^, ^34^, ^35^ Thus, it may be that the interaction(s) with other parameters in the traditional models have obscured this association.

Fluid related parameters identified by the ML model were IDWG, OH and TBW. OH and IDWG were statistically significant in the conventional logistic regression model and had similar direction as the advanced ML model. Patients with higher IDWG and higher OH had higher risk of mortality, thus emphasizing the essential role of fluid control in dialysis. The traditional model also suggests that the TBW is associated with lower mortality; however, it is important to note that this is not an important predictor (*p* = 0.1637) in the logistic regression model. The ML model on the other hand shows TBW is the 22nd most important factor suggesting that it may be important for a select group of patients where the data are available. So having a TBW measurement itself may decrease the risk due to a more targeted therapy being delivered with the BIS. We see similar results for all other BIS parameters, where high OH slightly increases the risk of death, but low OH is highly protective for those who have the data. For all these BIS measures, missing values are in the center suggesting they do not affect the risk and are missing at random.

Our ML model also showed patients with higher BMI had lower risk of mortality, which is consistent with previous findings.^36^, ^37^ Surprisingly, our logistic model found opposite signals with a small positive association in the risk of mortality with higher BMI. There were also contrasts between modeling techniques with higher RRF, where the ML model showed higher RRF was protective as expected, while the traditional model showed the risk of mortality slightly increased with increasing RRF. This result is likely the secondary to the imputation of missing values in traditional models. Advanced imputation techniques may have resulted in a different outcome. The inclusion of multiple covariates in a model can lead to biases if each associated parameter is interpreted causally often referred to as Table 2 fallacy.^38^ Thus, caution is warranted in the interpretation of specific associations in our models, since the goal of our analysis is predictive, rather than causal.^39^ For instance, inclusion in the model of any downstream variable in a causal pathway starting with RRF can potentially lead to collider bias, which can reverse the direction of expected associations.^40^

The partial dependence plot created using the SHAP values shows if bi‐modal interactions between parameters exist in more than one domain. Although this paper presents only two of these relations among the important domains from the top predictors common to both the models, other interactions between input parameters from other domains could be evaluated. Another limitation of the model is that it did not study the impact and interaction of the cardiovascular parameters such as blood pressure measurements. There may be many other potentially important parameters including comorbidity burden that could be evaluated, and optimization for specific parameters more readily available at specific providers could be considered. This model has only been tested on HD patients and may not be applicable to patients who are on peritoneal dialysis or other forms of home or incenter dialysis. Albeit death events were recorded during the provision of dialysis care worldwide in MONDO, the database did not capture data on patients who transitioned to other kidney replacement modalities (e.g., peritoneal dialysis, transplant), left the provider, or became lost to follow‐up and this could have the potential to introduce bias in the classification of survival. The model currently predicts death in the 3 years following the baseline period; however, the model could be designed to predict death at various other time periods. It is also important to note that in this analysis we have not considered interactions between the input parameters when developing the traditional models; however, it is possible that the performance of the traditional may have improved if we considered these interactions. Advanced ML model automatically disentangles these complexities of the relationship between input parameters. Many traditional models, when compared to ML models, assume linearity, and avoid known interactions. Thus, we may not be comparing the best traditional method with ML technique.^41^ ML models are often considered a black box that are difficult to interpret how the model arrives at its prediction.^42^ Since the goal of this paper is not only to study the risk factors associated with mortality but also to compare the outputs of the two modeling techniques, the ML model was not optimized for performance using hyperparameter tuning and this could yield further improvements.^43^ If fully optimized and implemented in clinical practice, ML models could provide a suitable method to help identify a patient's prognosis and drive pre‐emptive interventions and/or care planning efforts. Additionally, MONDO database is undergoing an update with most recent data and additional data points being captured. This proof‐of‐concept model can be scaled to adapt to additional input parameters or different follow‐up time periods.

## CONCLUSION

This paper demonstrates the successful use of ML and traditional statistical techniques in predicting mortality on a large cohort of international HD patients using clinical parameters collected consistently across regions. It shows how the risk factors compare between the ML techniques and conventional modeling techniques. If implemented in clinical practice, such models could be used to provide pre‐emptive care for HD patients.

## AUTHOR CONTRIBUTIONS

Design was performed by Sheetal Chaudhuri, Peter Kotanko, Len Usvyat, and Jeroen P. Kooman. Data Extraction and analysis were performed by Sheetal Chaudhuri, Jeroen P. Kooman, John Larkin, Murilo Guedes, Yue Jiao, Yuedong Wang, and Len Usvyat. The interpretation, drafting, and revision of this manuscript were conducted by all authors. The decision to submit this manuscript for publication was jointly made by all authors and the manuscript was confirmed to be accurate and approved by all authors.

## FUNDING INFORMATION

Analysis was supported by Fresenius Medical Care.

## CONFLICT OF INTEREST

Sheetal Chaudhuri is a student at Maastricht University Medical Center. Sheetal Chaudhuri, John Larkin, Yue Jiao, and Len Usvyat are employees of Fresenius Medical Care. Peter Kotanko is an employee of Renal Research Institute, a wholly owned subsidiary of Fresenius Medical Care. Sheetal Chaudhuri, John Larkin, Len Usvyat, and Peter Kotanko have share options/ownership in Fresenius Medical Care. Sheetal Chaudhuri, John Larkin, Len Usvyat, and Peter Kotanko are an inventor on patent(s) in the field of dialysis. John Larkin has received honorarium from The Lancet and is a guest editor on the Editorial Board of Frontiers in Physiology. Len Usvyat is a member on Privacy Analytics' Advisory Board. Peter Kotanko receives honorarium from Up‐To‐Date and is on the Editorial Board of Blood Purification and Kidney and Blood Pressure Research. Yuedong Wang and Murilo Guedes has no relevant conflicts of interest to disclose.
